# Irritable bowel syndrome: prevalence, risk factors in an adult Lebanese population

**DOI:** 10.1186/s12876-017-0698-2

**Published:** 2017-12-02

**Authors:** Rajaa Chatila, Mahmoud Merhi, Essa Hariri, Nada Sabbah, Mary E. Deeb

**Affiliations:** Gilbert and Rose-Marie Chagoury School of Medicine, Lebanese American University Medical Center-Rizk Hospital, P.O. Box 36, Byblos, Lebanon

**Keywords:** Irritable bowel syndrome, Rome III criteria, Alcohol, Cigarette smoking, Water pipe, Physical activity, Prevalence, Lebanon

## Abstract

**Background:**

Very few studies report on the prevalence of irritable bowel syndrome (IBS) and its correlates in the Middle East. This study investigated Irritable Bowel Syndrome (IBS) prevalence in a sample of Lebanese adult individuals and associated demographic and behavioral lifestyle factors.

**Methods:**

This is an observational population-based study. The target population is working Lebanese adults, eighteen-to-sixty five years old. The sample was selected from a convenience population of bank employees in different geographical areas in Lebanon. The study participants completed an anonymous self-administered questionnaire, to collect data on their socio-demographic, behavioral and life style characteristics, and diagnostic questions following Rome III criteria to assess IBS occurrence. The difference in IBS prevalence by socio-demographic characteristics, smoking, alcohol consumption, and physical activity was assessed by using the Chi-square test. Logistic regression adjusted odds ratios were used to investigate the association between risk factors and IBS.

**Results:**

Data was collected from 553 individuals and consisted of 52.8% females (mean age 35.9 years, SD = 11.9) and 47.2% males (mean age = 36.1 years, SD = 10.3). The prevalence of IBS in the study population according to Rome III criteria was 20.1%. The bivariate analysis indicated that being younger than 30 years old, a female, an ever water pipe smoker, an ever alcohol consumer are significantly associated with a higher prevalence of IBS. Educational level, cigarettes smoking and physical exercise were not significantly associated with IBS occurrence. The logistic regression adjusted odds ratio showed that females were 1.67 times more likely to have IBS than males (P˂ 0.05). The participants aged less than 30 years old were at a higher risk of having IBS (P˂ 0.01). Those who ever smoked waterpipe were 1.63 times more likely to have IBS than those who never smoked waterpipe (P˂ 0.05). Those who were ever alcohol drinkers were twice as likely to have IBS than never-drinkers (P˂ 0.01).

**Conclusion:**

New data on the high prevalence of IBS in an adult population in Lebanon has been reported. This is also the first study to investigate and show an association of waterpipe smoking and IBS. Further longitudinal studies are warranted to determine whether this association is causal.

**Electronic supplementary material:**

The online version of this article (10.1186/s12876-017-0698-2) contains supplementary material, which is available to authorized users.

## Background

Irritable bowel syndrome (IBS) is a functional gastrointestinal disorder characterized by abdominal pain and alterations in bowel habits [1]. The global prevalence of IBS is estimated to be 11.2%, and it is the most common functional gastrointestinal disease. IBS is not a life-threatening condition, yet people with IBS have a reduced quality of life that may affect their educational, social and occupational achievements [2]. The economic burden of IBS is substantial on the healthcare system too. The direct annual cost of diagnosing and treating IBS in the United States is estimated between $1.7 and $10 billion. The indirect costs in terms of absenteeism, workdays lost, disability will double the monetary figure estimated as direct costs [3].

There are several criteria for diagnosing IBS including Rome I, Rome II and Manning criteria, but the most widely used method is the Rome III criteria [4]. Thus, the use of different diagnostic criteria will affect the reported IBS prevalence worldwide. Studies using the Rome III criteria, report the prevalence of IBS in Western countries range from 10 to 20% [5]compared with 1 to 10% in the Asian countries [6]. The lowest reported rates were in Southeast Asia (7.0%) while the highest (21.0%) were in South America. The prevalence of IBS varies by socio-demographic factors, gender and age [7, 8]. In developed countries, women are 2-4 times more likely to develop IBS compared to men [7, 9]. IBS is more prevalent among adolescents and declines with age [10].The odds of having IBS are higher among those younger than 50 years compared to those older than 50 years of age [11–13]. Lifestyle factors such as smoking, alcohol consumption [14–16] and physical activity [17, 18] have also been linked to IBS. Moreover, IBS has been associated with psychological factors such as stress and anxiety [6, 8], as well as genetics factors where 33% of patients with IBS report a positive family history [19].

Epidemiologic studies assessing the prevalence of IBS and its correlates are lacking in the developing world and specifically in African and Middle Eastern countries, particularly in Lebanon. Lebanon is a Middle Eastern middle-income country with a population of about four million that experienced a protracted civil war for almost two decades from 1975 to 1990. A recent study among university students in Lebanon reports a prevalence of 20% using the Rome III criteria [17]. Thus, the current study aims to (1) estimate the prevalence of IBS in a Lebanese population based on a sample of employed adults and to (2) assess the behavioral risk factors, including smoking, waterpipe, alcohol use and physical inactivity associated with the disease.

## Methods

### Study design and recruitment of study participants

This is an observational, population-based study. The target population is Lebanese adults, eighteen-to-sixty five years old. The sample was selected from a convenience population of bank employees in Lebanon. The choice of bank employees was based on the rationale that they represent a significant percent of the private working force. Bank employees also provide a sufficient wide age range of a cohort of working adults in Lebanon. The selected banks were located in major cities across the country: Beirut, the capital, Tripoli and Byblos, in the North, Zahle and Chtaura in the Bekaa, and Saida, Tyre and Nabatieh in the South of Lebanon. All major banks in Beirut were contacted initially by e-mail to request the administration approval to conduct the survey among their employees in the main headquarters in the city of Beirut, as well as branches in other cities. The selected banks were representative of the Lebanese banking sector, as they constituted the major banks in the country. The majority (95%) of the contacted banks agreed to participate.

The data collection extended from January 2016 to April 2016. The employees were informed about the survey by the administration and asked for voluntary participation in the study. The questionnaires were distributed and collected by the study team during working hours (Additional file 1). The consenting participants were asked to return the filled questionnaire in a sealed envelope and deposited in an assigned box for confidentiality. The response rate in different banks varied from 70% to 80%. Bank employees were excluded if they had a history of Crohn’s disease, or treated for peptic ulcer diseases. A screening question was used to exclude the non-eligible participants. The research team approached the consenting bank employees and inquired: Have you ever been diagnosed with Crohn’s disease? Are you currently taking medication for peptic ulcer disease?. Any respondent who responded yes to any of these two questions was not eligible to participate in the study. All eligible employees in the selected banks completed a fifteen minutes anonymous self-administered questionnaire. The questionnaire included a consent form on its cover page, all participants completing the questionnaire were considered to be indirectly providing their informed consent. The Lebanese American University Institutional Review Board (IRB) committee reviewed and approved the bank request formal letter as well as the study questionnaire.

Data to ascertain prevalence and diagnosis of IBS were based on the Rome III criteria for Functional Gastrointestinal Disorders. IBS is defined as recurrent abdominal pain or discomfort in the last three months for at least 3 days per month, associated with at least two of the following: relief after defecation; changes in bowel movement frequency, and occurrence of symptoms associated with changes in stool form [20]. The questionnaire collected data on socio-economic, demographic and behavioral characteristics (sex, age, education and regional distribution), smoking patterns (cigarettes, waterpipe, cigars and pipe), alcohol consumption physical activity and food intolerance.

### Sample size

The sample size calculation based on the probability that the prevalence of IBS is 20% in the population and the error in the estimate of ±3.4% with a 95% confidence interval yielded a required sample size of 532 individuals.

### Statistical analysis

The data was analyzed using the Statistical Package for Social Sciences (Version 23.0. IBM Corporation, Arnouk, USA). The association between IBS and socio-demographic characteristics, smoking, alcohol consumption, physical activity and food intolerance was assessed by Chi-square. Logistic regression was used to predict the independent association of demographic factors, smoking, alcohol consumption, and physical activity on the odds of having IBS. A *p*-value less than 0.05 indicated statistical significance.

## Results

The total number of questionnaires collected was 612 but some had to be discarded due to incomplete information on IBS and other characteristics. The final sample size was 553 individuals and consisted of 52.7% females and 47.3% males. The mean age among the females was 35.9 years (SD =11.9) and males 36.1 years (SD =10.3). The majority of the sample had a university degree 65.3%, 17.3% completed higher studies and 17.4% had either secondary education or a technical school degree, (Table 1).

**Table 1:** Distribution of participants by selected characteristics

Characteristics	N	% of Total
Gender		
Male	261	47.2
Female	292	52.8
Age (years)^a^		
18-30	212	40.8
31-40	142	27.4
41-50	109	21.0
50- 65	56	10.8
Bank Location		
Beirut	143	25.9
Bekaa	46	8.3
Mount Lebanon	54	9.8
North Lebanon	126	22.8
South Lebanon	184	26.2
Educational level^a^		
Completed Secondary	67	12.3
Technical School	28	5.1
University Degree	356	65.4
Higher Studies	94	17.2

^a^Totals do not add up to 553 due to missing data

(Table 1 to be inserted here)

### Prevalence and bivariate association of IBS by risk factors

The prevalence of IBS in the study population according to Rome III criteria was 20.1%. It varied by socio-demographic and lifestyle factors. The prevalence of IBS was higher among females (22.9%) compared to males (16.9%)(P˂0.05). Age was negatively related to IBS, study participants older than 30 years reported less IBS than those younger than 30 years of age. (P˂ 0.01). No significant difference was observed with respect to the educational level of the respondents, (Table 2).

**Table 2:** Distribution of IBS prevalence by respondent characteristics. Irritable Bowel Syndrome

Characteristics	Yes		No			
	N	%	N	%	Total N	P-value
Sex	111	20.1	442	79.9	553	
Male	44	16.9	217	83.1	261	0.046
Female	67	22.9	225	77.1	292	
Education level*						
Secondary/Technical	14	14.7	81	85.3	95	
University Degree	73	20.5	283	79.5	356	
Higher Studies	24	25.5	70	74.5	94	0.182
Cigarettes Smoking						
Never	57	18.4	252	81.6	309	
Ever	54	22.1	190	77.9	244	0.167
Waterpipe Smoking						
Never	49	16.6	246	83.4	295	
Ever	62	24.0	196	76.0	258	0.019
Alcohol Consumption ^a^						
Never	55	16.1	286	83.9	341	0.002
Ever	56	26.5	155	73.5	211	
Physical Activity ^a^						
Yes	71	19.9	285	79.6	356	0.49
None	40	20.4	156	80.1	196	
Food Intolerance^a^						
Yes	10	35.7	18	64.3	28	
No	101	19.3	422	80.7	523	0.037

^a^Totals do not add up to 553 due to missing data

(Table 2 to be inserted here)

### Cigarettes smoking

The prevalence of current cigarette smoking in the total sample was 31.3%, with 12.8% being past smokers, and the remaining 55.9% having never smoked. The prevalence of cigarette smoking was higher (P˂ 0.001) among males (56.3%) compared to females (33.2%). Smoking was more prevalent (P˂ 0.003) among those who had a secondary education or a technical school degree (57.9%) compared to those with a university degree or higher studies 42.4% and 39.4% respectively. No significant difference in IBS prevalence was observed between those who ever smoked and those who never smoked cigarettes, (Table 2).

### Waterpipe smoking

The prevalence of current waterpipe smoking was 36.6%, 10.1% were past smokers, while 53.3% never smoked. No sex or educational level differential was noted among waterpipe smokers. Table 2 show that IBS occurred more frequently among ever waterpipe smokers (24.0%) than those who never smoked water pipe (16.6%) (P˂0.01).

### Alcohol consumption

The prevalence of reported current alcohol consumption was 31.4% with 6.9% being past-drinkers and 61.7% never consumed alcohol. Males reported a greater (P˂0.01) alcohol consumption (47.6%) compared to females (34.8%). Alcohol consumption was more prevalent(P˂0.0001) among those who had a university or completed higher studies (58.2%) compared to those with secondary education or technical school (38.1%). Table 2 showed that IBS was more prevailing (P˂0.001) among alcohol drinkers (26.5%) compared to never alcohol drinkers (16.1%).

### Physical activity

The prevalence of physical activity was 63.9%, where 24.4% reported exercising less than once per week, 25.5% exercised 2 to 3 times per week and 14.0% exercised more than 3 times per week. A higher proportion of males (P˂0.0001) were physically active (77.9%) compared to females (53.8%), while no difference in activity level was noted between those with different levels of education. There was no change in IBS occurrence by reported physical activity, (Table 2).

### Food intolerance

The respondents were also asked to report if they experience any food intolerance. Those who reported experiencing food intolerance (*n* = 28) had a greater (P˂ 0.05) occurrence of IBS (35.7%) compared to those who did not report food intolerance (19.3%).

### Logistic regression analysis

Table 3 describes the adjusted odds ratio of IBS correlates and their confidence intervals. The logistic regression showed that the odds of having IBS are 1.69 times higher for females compared to males (P˂0.05). A younger age of less than 30 years old, was associated with a higher odds of IBS occurrence 1.80 than those older than 30 years old. Those who ever smoked water pipe were 1.63 times more likely to have IBS than those who never smoked water pipe (P˂0.05). Alcohol consumers were twice as likely to have IBS compared to non-alcohol consumers(P˂0.05). Physical exercise and cigarette smoking were not significantly associated with having IBS.

**Table 3:** Logistic Regression of IBS occurrence by demographic and life style risk factors

	*P*-value	Adjusted Odds Ratio	(95% Confidence Interval)
Gender			
Female	0.028	1.691	(1.059-2.699)
Male		1.00	
Waterpipe Smoking			
Ever	0.034	1.630	(1.038-2.599)
Never		1.00	
Physical Activity			
Yes	0.645	0.920	(0.550-1.448)
None		1.00	
Alcohol Consumption			
Ever	0.002	2.064	(1.309-3.254)
Never		1.00	
Age (years)			
≤ than 30	0.011	1.802	(1.147-2.831)
› than 30		1.00	

(Table 3 to be inserted here)

## Discussion

### Key findings

This study assessed the prevalence of IBS among a sample of Lebanese employees as well as major lifestyle associations. The prevalence of 20.1% among the sampled adult participants was comparable to two studies of university students, one in Lebanon [17] and a recent one in neighboring Syria [21]as well as estimates reported in developed countries [6]. However the occurrence of IBS was much higher than what has been reported in the West [2]. The reported sex and age differential among those with IBS was consistent with results reported in the literature [7, 8]. Various hypotheses have been proposed to explain the higher prevalence of IBS in females, i.e. higher serotonin synthesis in the brain [22],female sex hormones’ effect on gastrointestinal motility [23], and probable association of IBS with an anti-nociceptive mechanism diminishing pain related to pelvic events such as pregnancy and delivery [24].The association of IBS with educational level is inconsistent in the reported literature, where some studies show a higher prevalence of IBS among educated compared to less educated [25–27].Gwee et al. [28] reported that IBS is significantly more prevalent among those with more than 6 years of post-secondary education. Yet, lower education was associated with a higher IBS prevalence in one study [29]. There was minimal variability in the educational level of the participants in our study; therefore such a statistical association could not be analyzed adequately.

### Behavioral (lifestyle)risk factors

Individuals who consumed alcohol in our study were twice as likely to suffer from IBS compared to those who did not. The association between alcohol consumption and IBS has been inconsistent in the literature, some studies report no effect [14, 15], while others show that alcohol consumption was associated with a higher IBS prevalence [16]. Certainly, more studies are required to reach a better understanding of the association between alcohol and IBS.

Our study is the first to investigate the relationship between IBS and waterpipe smoking. Waterpipe smokers were found to have significantly more IBS compared to non-smokers. Waterpipe smoking (Hookah or Shisha) is becoming popular in coffee shops across the Middle East and North Africa and its prevalence in Lebanese youth (13-15 years) has reached 64.5% of males and 54.6% of females; whereas its prevalence in Lebanese adults (18+ years) is about 25.8% for males and 23.3% for females. Unfortunately, Lebanese women have the highest female reported water pipe smoking rate in the region [30]. This social habit seems to have spread to Western countries namely in Europe [31, 32].

Cigarette smoking was significantly more prevalent in males (56.3%) compared to females (33.2%), yet no sex difference was noted in waterpipe smoking. One probable explanation for this difference in sex prevalence seen in cigarette but not waterpipe smoking can be related to the wide social acceptance of waterpipe in the Lebanese culture and the limited knowledge about its risks.

Physical activity has been shown to be an effective measure in relieving gas-related symptoms, and is currently recommended for people who suffer from abdominal bloating as it improves impaired gas clearance related to altered small bowel activity [33].Although Costanian et al. [17], Kim et al. [18] and others [34]have consistently shown higher IBS prevalence in those with low physical activity and that activity improved IBS symptoms, our study showed that physical activity was not significantly associated with IBS occurrence in this adult sample of the Lebanese population.

The role of food intolerance in provoking or exacerbating IBS symptoms have been well described and constitute the basis of the low FODMAP diet [35] . The significant relationship observed in our study of IBS in patient reporting food intolerance, is well established and in agreement with what has been reported in the literature. However, the respondents were not asked what type of food induces the food intolerance, which is a study limitation.

### Study limitation

There are several limitations that should be noted in this study. The data was collected through self-administered questionnaires that may lead to a higher percentage of incomplete data than interview surveys. Selection bias is also possible as we limited the study population to bank employees that might already have a better socio-economic status and educational level compared to the general Lebanese population. This was evident in the finding that 65% of our sample had a university degree compared to 20% in the general population in Lebanon. Moreover, other factors known to be associated with IBS were not addressed, such as depression and anxiety. In addition, the amount of tobacco use, alcohol consumption, and water pipe use was not quantified.

## Conclusions

In conclusion, the prevalence of IBS in our sample of employed adults in the banking businesses was high, reaching the upper limit of worldwide prevalence (20%). Given the impact of IBS on the quality of life of those afflicted with the disease, a better understanding of the prevalence and associated socioeconomic and behavioral risk factors among the Lebanese is needed. This is one of the first studies to estimate the prevalence of IBS and its association with lifestyle risk factors (physical activity, waterpipe smoking and alcohol consumption) in an adult subset of the Lebanese population. With respect to waterpipe smoking and its association with IBS, a longitudinal cohort study, controlling for major confounders such a stress is warranted to establish causality of this observed correlation. Future studies would open the door towards an improved understanding of IBS complex GI pathology, and allow a healthier management geared towards the psychological and lifestyle factors related to IBS.

## Additional file

Additional file 1: Bloating Questionnaire. (DOCX 98 kb)

## Abbreviations

IBS: Irritable bowel syndrome
P: *P*-value
SD: Standard deviation
