# Supplementary material for: Irritable bowel syndrome: prevalence, risk factors in an adult Lebanese population
Source: BMC Gastroenterol. 2017 Dec 2;17:137. doi: 10.1186/s12876-017-0698-2 (PMC5712083; doi:10.1186/s12876-017-0698-2)
Supplement: Additional file 1: — Bloating Questionnaire. (DOCX 98 kb) [file 12876_2017_698_MOESM1_ESM.docx]

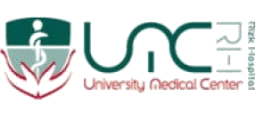

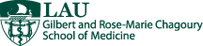


**Informed Consent:**

You will be asked to fill out a short questionnaire, which is part of an epidemiological study that aims at investigating a correlation between Nargileh smoking and abdominal bloating. Please feel free to stop at any time it you have any inquiries regarding the study.

Your privacy and the confidentiality of the information you provide will be strictly respected in all published and written data analysis resulting from this study. The study is strictly anonymous.

It should take approximately *15 minutes* of your time.

Your participation is on a voluntary basis and you have the right to withdraw your consent or discontinue participation at any time without penalty.

At no instance will you be asked to reveal any personal information. Participation in this study involves no major risks whatsoever, be it physical or emotional. The study is not directly beneficial to the participants; however, it will be of scientific and medical value. You will receive no incentive or payment for your participation. *Your refusal to participate will not result in any penalty or loss of benefits to which you are otherwise entitled to*

*The research intends to abide by all commonly acknowledged ethical codes. You agree to participate in this research project by filling the following questionnaire. If you have any questions, please ask the research team listed at the beginning of this questionnaire. Thank you for your time.*

*If you have any questions, you may contact:*

| *Name (PI)* | *Phone number* | *Email address* |
| --- | --- | --- |
| Dr. Rajaa Chatila | 03 539849 | [rajaa.chatila@lau.edu.lb](mailto:rajaa.chatila@lau.edu.lb) |
| Dr. Mary Deeb | 03 477551 | mary.deeb@lau.edu.lb |

*If you have any questions about your rights as a participant in this study, or you want to talk to someone outside the research, please contact the:*

*IRB Office,*

*Lebanese American University*

*3^rd^ Floor, Dorm A, Byblos Campus*

*Tel: 00 961 1 786456 ext. (2332)*

**Section I:** Demographics

**I.D.**

**Address:**

1. Beirut
2. Beirut Suburbs
3. Mount Lebanon
4. South
5. North
6. Nabatiyeh
7. Bekaa

**Q1. Sex**:

1. Female

2. Male

**Q2. Age** _____ (years)

**Q3. Educational Level**:

1. Illiterate
2. Completed primary school

1. Completed secondary school
2. Technical School
3. University Degree
4. Graduate Studies

**Section II:** Cigarette/Cigars/Pipe Smoking

**Q4.1 Have you ever smoked cigarettes?**

1. Never (go to Q.4.7)
2. Yes, and still smoking (go to Q.4.4)
3. Yes and stopped

**Q4.2 When did you stop smoking cigarettes?** No. of years ago _________

0. Not applicable

**Q4.3 Why did you stop smoking cigarettes?**

0. Not applicable

1. A health professional told me to, but not for medical reasons

2. A health professional told me to, for medical reasons

3. Religious/moral reasons

4. Too expensive

5. Better for my health

**Q4.4 At what age did you start smoking cigarettes?** Age ___________

0. Not applicable

**Q4.5 How many did you/do you smoke per day?** ____ box(es) or ____ cigarette

0. Not applicable

**Q.4.6:** *Questions below refer to the last time the participant smoked cigarettes or in the past week.*

|  | **Yes** | **No** |
| --- | --- | --- |
| **Q 4.6.1 Do you regularly experience a bloating sensation directly** **after cigarettes smoking?** |  |  |
| **Q 4.6.2. Do you regularly experience visible expansion directly** **after cigarettes smoking?** |  |  |
| **Q 4.6.3. Do you experience any vomiting directly after cigarettes smoking?** |  |  |
| **Q 4.6.4. Do you experience any light-headedness directly after cigarettes smoking?** |  |  |
| **Q 4.6.5. Do you experience any nausea directly after cigarettes smoking?** |  |  |

**Q4.7 Have you ever smoked cigars?**

1. Never (go to Q 4.13)

2. Yes, and still smoking (go to Q 4.10)

3. Yes, and stopped

**Q4.8 When did you stop smoking cigars?** No. of years ago _____________

0. Not applicable

**Q4.9 Why did you stop smoking cigars?**

0. Not applicable

1.A Health professional told me to, but not for medical reasons

2.A Health professional told me to, for medical reasons

3. Religious/moral reasons

4. Too expensive

5. Better for my health

**Q4.10 At what age did you start smoking cigars?** Age ___________

0. Not applicable

**Q4.11 How many did you/do you smoke per day?** Number ___________

0. Not applicable

**Q.4.12:** *Questions below refer to the last time the participant smoked cigar or in the past week.*

|  | **Yes** | **No** |
| --- | --- | --- |
| **Q 4.12.1 Do you regularly experience a bloating sensation directly** **after cigar smoking?** |  |  |
| **Q 4.12.2. Do you regularly experience visible expansion directly** **after cigar smoking?** |  |  |
| **Q 4.12.3. Do you experience any vomiting directly after cigar smoking?** |  |  |
| **Q 4.12.4. Do you experience any light-headedness directly after cigar smoking?** |  |  |
| **Q 4.12.5. Do you experience any nausea directly after cigar smoking?** |  |  |

**Q4.13 Have you ever smoked pipe?**

1. Never (go to Q 5.1)

2. Yes, and still smoking (go to Q 4.16)

3. Yes, and stopped

**Q4.14 When did you stop smoking pipe?** No. of years ago _____________

0. Not applicable

**Q4.15 Why did you stop smoking pipe?**

0. Not applicable

1.A Health professional told me to, but not for medical reasons

2.A Health professional told me to, for medical reasons

3. Religious/moral reasons

4. Too expensive

|  | **Yes** | **No** |
| --- | --- | --- |
| **Q 4.18.1 Do you regularly experience a bloating sensation directly** **after pipe smoking?** |  |  |
| **Q 4.18.2. Do you regularly experience visible expansion directly** **after pipe smoking?** |  |  |
| **Q 4.18.3. Do you experience any vomiting directly after pipe smoking?** |  |  |
| **Q 4.18.4. Do you experience any light-headedness directly after pipe smoking?** |  |  |
| **Q 4.18.5. Do you experience any nausea directly after pipe smoking?** |  |  |

5. Better for my health

**Q4.16 At what age did you start smoking pipe?** Age ___________

0. Not applicable

**Q4.17 How many did you/do you smoke per day?** Number ___________

0. Not applicable

**Q.4.18:** *Questions below refer to the last time the participant pipe or in the past week.*

**Section III:** Hubble-Bubble

**Q5.1 Have you ever smoked Hubble-Bubble (HB)?**

1. Never (go to Q 6)

2. Yes, and still smoking (go to Q 5.5)

3. Yes, and stopped

**Q5.2 When did you stop smoking HB?** No. of years ago _____________

0. Not applicable

**Q5.3 Why did you stop smoking HB?**

0. Not applicable

1.A Health professional told me to, but not for medical reasons

2.A Health professional told me to, for medical reasons

3. Religious/moral reasons

4. Too expensive

5. Better for my health

**Q5.4 At what age did you start smoking HB?** Age ___________

0. Not applicable

**Q5.5 How many did you/do you smoke per day/week/month?** Number ___________

0. Not applicable

**Q5.6 Do you retain the smoke most of the time?**

- - - 1. Yes
      2. No

**Q5.7 Duration of smoking per session:**

1. Less than ½ hour
2. Less than 1 hour
3. More than 1 hour

**Q5.8 Any preferable type or flavor? ________**

1. Ajami Yes  No
2. M3assal Yes  No

**Q.5.9:** *Questions below refer to the last time the participant smoked HB or in the past week.*

|  | **Yes** | **No** |
| --- | --- | --- |
| **Q5.9.1 Do you regularly experience a bloating sensation directly** **after HB smoking?** |  |  |
| **Q5.9.2. Do you regularly experience visible expansion directly** **after HB smoking?** |  |  |
| **Q5.9.3. Do you experience any vomiting directly after HB smoking?** |  |  |
| **Q5.9.4. Do you experience any light-headedness directly after HB smoking?** |  |  |
| **Q5.9.5. Do you experience any nausea directly after HB smoking?** |  |  |

**Q5.9.6 Do you smoke while you are eating or after you finish your meal?**

- - - 1. Yes, I smoke usually while eating
      2. Yes, I smoke usually after I finish my meal
      3. Yes, but no regular pattern
      4. No

If yes:

- **Q5.9.6.1** Do you experience a bloating sensation?

1. Yes  2. No

- **Q5.9.6.1** Do you experience visible expansion?

1. Yes  2. No

**Section IV:** Alcohol

**Q6.1 Have you ever drunk alcohol?**

1. Never (go to Q 7)

2. Yes, and still drinking (go to Q 6.4)

3. Yes, and stopped

**Q6.2 When did you stop drinking alcohol?** No. of years ago _____________

0. Not applicable

**Q6.3 Why did you stop drinking alcohol?**

0. Not applicable

1.A Health professional told me to, but not for medical reasons

2.A Health professional told me to, for medical reasons

3. Religious/moral reasons

4. Too expensive

5. Better for my health

**Q6.4 At what age did you start drinking alcohol?** Age ___________

0. Not applicable

**Q6.5 How often did you/do you have a drink?**

0. Not applicable

1. Not every week

2. 1-2 times a week

3. >2 times a week

4. On occasion

**Q 6.6 How many drinks you have in each session?**

- - 1. 1-2
    2. 3-4
    3. 5-6
    4. 7-9
    5. More than 9

**Q 6.7 What type of** **alcoholic drink you do you take?**

1. Beer
2. Whiskey
3. Wine
4. Vodka
5. Spirits(Arak)
6. Others _____________

**Q 6.8 Why did you start drinking alcohol?**

0. Not applicable

1. Doctor's advice

2. I think it is healthy

3. War

4. Social reasons

5. Other, specify

**Q 6.9:**

|  | **Yes** | **No** |
| --- | --- | --- |
| **Q6.9.1 Do you regularly experience a bloating sensation directly** **after alcohol drinking?** |  |  |
| **Q6.9.2. Do you regularly experience visible expansion directly** **after alcohol drinking?** |  |  |
| **Q6.9.3. Do you experience any vomiting directly after alcohol drinking?** |  |  |
| **Q6.9.4. Do you experience any light-headedness directly after alcohol drinking?** |  |  |
| **Q6.9.5. Do you experience any nausea directly after alcohol drinking?** |  |  |

**Section V:** Medical History

**Q7. Have you ever suffered from any of the following conditions?**

|  | | **Yes** | **No** |
| --- | --- | --- | --- |
| **Diabetes Mellitus** |  |  |  |
| **Hypertension (High blood pressure)** |  |  |  |
| **Coronary Artery Disease (Heart Disease)** |  |  |  |
| **Stroke (Brain Attack)** |  |  |  |
| **Asthma** |  |  |  |
| **Dyslipidemia (High Cholesterol)** |  |  |  |
| **Mental illness** |  |  |  |
| **Food Intolerance (Specify):** |  |  |  |
| **Other (specify):** |  |  |  |

**Q8. Have you ever had an abdominal surgery?**

1. **Yes**
2. **No**

**Q8.1 If yes please specify:**

1. Cholecystectomy (Gall Bladder removal)
2. Appendectomy (removal of the Appendix)
3. Colectomy (Colon Resection)
4. Hernia repair
5. Others  ….………………………………..

**Q9. Are you currently taking any medication?**

1. Yes
2. No

**Q9.1 If yes, please list them below:**

**Q 10. Do you engage in some form of exercise on a regular basis?**

0. Don't exercise (go to Q )

1. Walking

2. Jogging

3. Swimming

4. Tennis

5. Aerobics

6. Others _______

**Q 10.1 How often do you exercise?**

1. Never
2. Less than 2 times per week
3. 2-3 times per week
4. More than 3 times per week

**Q 11. Do you experience any of the following?**

|  | **Yes** | **No** |
| --- | --- | --- |
| **Q 11.1 Recurrent feeling of bloating at least 3 days/month in the last 6 months?** |  |  |
| **Q 11.2 Recurrent visible abdominal expansion at least 3 days/month in the last 6 months?** |  |  |
| **Q 11.3 Symptoms of bloating or visible expansion in the last 6 months?** |  |  |
| **Q 11.4 Bothersome epigastric pain (upper abdominal) or burning sensation for the last 6 months?** |  |  |
| **Q 11.5 Bothersome repetitive belching (burping) at least several times a week for the last 6 months?** |  |  |
| **Q 11.6 Any unintentional weight loss in the last 6 months?** |  |  |
| **Q 11.7 Recurrent nausea and vomiting in the past 6months?** |  |  |
| **Q 11.8 Personal history of rectal bleeding or passage of pus per rectum in the past?** |  |  |

**Q 11.9 Abdominal pain or discomfort at least 3 days/month in the last 6 months?**

- 1. Yes
  2. No

If yes:

- **Q 11.9.1 Does your abdominal pain or discomfort improve with defecation?**
  1. Yes
  2. No
  3. Sometimes
- **Q 11.9.2 Is your abdominal pain or discomfort associated with a change in frequency or form (appearance) of stool “constipation or diarrhea”**
  1. Yes
  2. No
- **Q 11.9.3 Bothersome postprandial (after-meal) fullness or early satiation occurring after ordinary sized meals at least several times per week for the last 6 months?**
  1. Yes
  2. No

**~ Thank you for participating in our survey ~**
